# Presence of a Polymicrobial Endometrial Biofilm in Patients with Bacterial Vaginosis

**DOI:** 10.1371/journal.pone.0053997

**Published:** 2013-01-08

**Authors:** Alexander Swidsinski, Hans Verstraelen, Vera Loening-Baucke, Sonja Swidsinski, Werner Mendling, Zaher Halwani

**Affiliations:** 1 Charité Hospital, CCM, Laboratory for Molecular Genetics, Polymicrobial Infections and Bacterial Biofilms and Department of Medicine, Gastroenterology, Universitätsmedizin Berlin, Berlin, Germany; 2 Department of Obstetrics and Gynaecology, Ghent University, Ghent, Belgium; 3 Department of Microbiology, Labor Berlin, Neukölln, Berlin, Germany; 4 German Centre for Infections in Obstetrics and Gynaecology, Wuppertal, Germany; 5 DRK Kliniken Berlin Westend, Klinik für Gynäkologie und Geburtshilfe, Berlin, Germany; Columbia University, United States of America

## Abstract

**Objective:**

To assess whether the bacterial vaginosis biofilm extends into the upper female genital tract.

**Study Design:**

Endometrial samples obtained during curettage and fallopian tube samples obtained during salpingectomy were collected. Endometrial and fallopian tube samples were analyzed for the presence of bacteria with fluorescence-in-situ-hybridisation (FISH) analysis with probes targeting bacterial vaginosis-associated and other bacteria.

**Results:**

A structured polymicrobial *Gardnerella vaginalis* biofilm could be detected in part of the endometrial and fallopian tube specimens. Women with bacterial vaginosis had a 50.0% (95% CI 24.0–76.0) risk of presenting with an endometrial *Gardnerella vaginalis* biofilm. Pregnancy (AOR  = 41.5, 95% CI 5.0–341.9, p<0.001) and the presence of bacterial vaginosis (AOR  = 23.2, 95% CI 2.6–205.9, p<0.001) were highly predictive of the presence of uterine or fallopian bacterial colonisation when compared to non-pregnant women without bacterial vaginosis.

**Conclusion:**

Bacterial vaginosis is frequently associated with the presence of a structured polymicrobial *Gardnerella vaginalis* biofilm attached to the endometrium. This may have major implications for our understanding of the pathogenesis of adverse pregnancy outcome in association with bacterial vaginosis.

## Introduction

Bacterial vaginosis is the most common form of vaginal infection, typically causing vulvovaginal discomfort, including vaginal discharge, malodour and vulvar irritation [1]. A vast body of evidence shows however that the pathogenic effects of bacterial vaginosis are not confined to the lower genital tract. In particular, bacterial vaginosis is strongly associated with late foetal loss [2] and preterm birth [3], presumably due to an ascending genital tract infection pathway, though the precise mechanisms involved remain elusive. Among patients with infertility, substantially higher rates of bacterial vaginosis have been documented [4], as well as an increased risk of early pregnancy loss associated with bacterial vaginosis [5].

The microbiological correlate of bacterial vaginosis has been shown to involve a dense, highly structured polymicrobial biofilm, primarily consisting of *Gardnerella vaginalis*, strongly adhering to the vaginal epithelium [6]. This in turn might explain the recurrent nature of this condition [7], as has been shown for other biofilm-associated infections. As the vaginal microbiota seem to correspond with the perianal microbiota [8], we have previously investigated whether the bacterial vaginosis biofilm might extend to the perianal region, but failed to document such a continuum [9]. In the present study, we obtained endometrial and fallopian tube tissue samples and assessed whether the bacterial vaginosis biofilm might extend into the upper female genital tract. Andrews *et al* previously reported on a consistent association between bacterial vaginosis and an increased frequency of endometrial colonization with bacterial vaginosis-associated microorganisms [10].

## Materials and Methods

### Patients

Patients were recruited at the Gynaecology Department of the Charité Hospital, Berlin, Germany through written and oral informed consent. The Charité – Universitätsmedizin Berlin Ethics Committee (http://ethikkommission.charite.de/en/) specifically approved this study. Patients were considered eligible, if they were scheduled to have a curettage or a laparoscopic salpingectomy, regardless of indication. In the morning just prior to surgery, patients were asked to provide a first void urine sample, which was immediately fixated in Carnoy solution and transported to the laboratory for further analysis. Assessment of the presence or absence of bacterial vaginosis was based on FISH analysis of bacteria attached to desquamated vaginal epithelial cells in the urine sediment [11]. Even so this method is relatively new, we have recently shown that compared to Nugent scoring, FISH based analysis has an accuracy for the diagnosis of BV of 0.94 [95% CI 0.86–0.98] (sensitivity 0.83 [95% CI 0.51–0.97], specificity 0.97 [95% CI 0.87–0.99], PPV 0.83 [95% CI 0.51–0.97], and NPV 0.97 [95% CI 0.87–0.99] (Verstraelen & Swidsinski, in press).

### Desquamated epithelial cells of the vagina in urine sediments

Two millilitres of urine were mixed with 8 ml of Carnoy fixative in a 15 ml Falcon tube. An aliquot of 1.5 ml urine/Carnoy mix was centrifuged in a 1.5 ml Eppendorf tube for 6 minutes at 6,000 G. The sediment was decanted; the tube was filled with 1 ml of Carnoy solution and left at room temperature. After 1–5 minutes, the sediment was centrifuged once more (6 min/9,000 G), decanted, 50 µL Carnoy solution was added, and then stored at 4°C.

### Analysis of urine sediments

**Figure 1:**
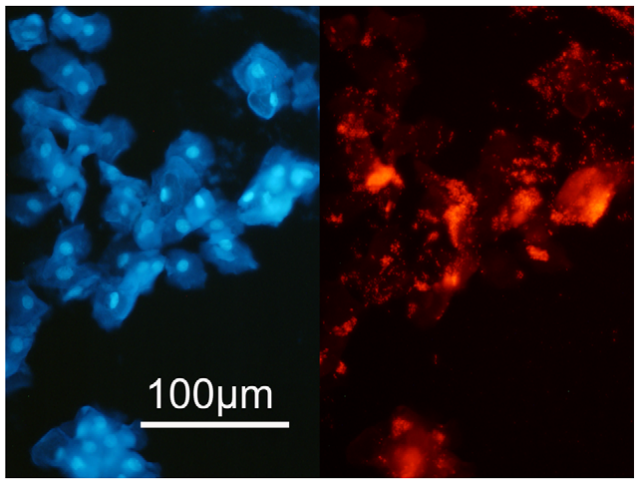
*Gardnerella* dominated polymicrobial biofilm cover the vaginal cells in urine. Urine sediment from a woman with a *Gardnerella* dominated polymicrobial vaginal biofilm. Left panel: DAPI stain of urine sediment with desquamated epithelial cells, right panel: the same microscopic field hybridized with *Gard* C5 FISH probe, dark red fluorescence at magnification ×400.

A 5×5 mm quadrant area of hybridization was marked with a PAP Pen on a SuperFrost plus glass slide. The Carnoy fixated urine sediment was vortexed; 5 µl aliquots were pipetted within the area of hybridization and dried for 30 minutes at 50°C just prior to the hybridization. Five microliters of the final aliquot were used for single hybridizations and represented 30 µl of the initial urine volume. Concentrations of epithelial cells within 5×5 mm area of hybridization (30 µl of sample volume) were calculated and converted to numbers of epithelial cells per ml of urine. The maximal and mean numbers of adherent bacteria per epithelial cell were determined. The overall concentrations of adherent bacteria in the urine resulted from multiplication of mean number of bacteria per epithelial cell with the concentration of epithelial cells per ml of urine.Adherent *Gardnerella* biofilms were recognized on typical appearance of structured *Gardnerella* dominated polymicrobial adherent biofilms attached to desquamated vaginal epithelial cells (see Figure 1). The assessor (AS) was blinded to patient status and other results of the study.

### Tissue samples

Surgically removed tissue samples were divided into two portions, the larger fragment was send to the pathologist and the smaller sample reduced to a size of maximal 1×1 cm and fixated in 25 ml of Carnoy solutions for at least 24 hours. Carnoy fixated material was then processed and embedded into paraffin blocks using standard techniques. Four µm sections were placed on SuperFrost slides (R. Langenbrinck, Emmendingen, Germany) for FISH studies [6], [7].

### Fixation

Fixation was performed with modified non-aqueous Carnoy solution (6/6/1 vol. ethanol/glacial acetic acid/chloroform). The fixated material could be stored in Carnoy at room temperature up to 6 months.

### FISH

Bacteria were assessed in a multi-colour analysis using a mix of specific and universal FISH probes stained with Cy3 (orange fluorescence), FITC (green fluorescence), Cy5 (dark red fluorescence) and DAPI counter stain (blue fluorescence) according to previously described protocols [6], [7].

The *GardV*, *Ato, Lab, Bac 303, Ebac*, and *Eub 338* probes were applied to each sample thereby targeting *Gardnerella, Atopobium, Lactobacillus, Bacteroides/Prevotella, Enterobacteriaceae*, and bacteria from the *Eubacteria* cluster.

Nikon e600 fluorescence microscope, Nikon DXM1200F camera and accompanying software (Nikon, Tokyo, Japan) were used. The enumeration of bacteria was performed when hybridization signals were clear and morphologically distinguishable as bacterial cells by at least triple colour identification with universal and group-specific FISH probes and DAPI stain, with absence of cross-hybridization with taxonomically unrelated probes [6], [7].

The conversion of the numbers within defined microscopic arears to concentrations of bacteria per ml was based on the calculation that a 10-µl sample with a cell concentration of 10^7^ cells per ml has 40 cells per average microscopic field at a magnification of ×1000, the details of conversion were previously published [6], [7].

### Sample size and statistics

Since this was an explorative, observational study, no power or sample size calculations were made. Rather a convenience sample of consecutive patients scheduled for curettage or salpingectomy for non-infectious indications was used. Ratios of categorical variables were compared with ordinary chi-square tests or with Fisher exact tests when appropriate. Strength of association was calculated in a bivariate analysis as ordinary odds ratios (OR). Ninety-five percent confidence intervals and P values to the confidence intervals are reported with ORs. Adjusted odds ratios (AOR) were obtained from a stepwise likelihood ratio-based binary logistic regression model. For any reported measure, statistical significance was accepted, as the 2-tailed probability level of <.05. All statistical analyses were performed using the statistical software package IBM SPSS Statistics v19.

## Results

Endometrial specimens were obtained from 46 patients, including 19 patients who underwent curettage for missed abortion and 27 patients who had a uterine scraping for other indications, including dysfunctional bleeding and uterine polyposis. Fallopian tube specimens were obtained from 22 women undergoing fallopian tube resection for various non-infectious indications, involving one patient with an extra-uterine pregnancy.

Bacterial vaginosis was found in eight women with missed abortion (8/19), in six women undergoing curettage in the absence of pregnancy (6/27), and in four women undergoing fallopian tube resection (4/22). Hence, the overall prevalence of bacterial vaginosis was 26.5% in this study cohort (see Table 1 and Table 2). Pregnant women (primarily women with missed abortion and one woman with an extra-uterine pregnancy) were significantly more likely to present with bacterial vaginosis (9/20) as compared to non-pregnant women (9/48) (OR  = 3.6, 95% CI 1.1–11.1, *P* = 0.02) or when compared to randomly selected pregnant women from the control population (12/72) in a previous study [11].

**Table 1:** Occurrence of bacteria on the endometrium in women with and without bacterial vaginosis.

*Presence of bacterial vaginosis*	*Presence of bacteria on the endometrium*					
	*Missed abortion (n = 19)*			*Other indications (n = 27)*		
	*endometrial bacteria*			*endometrial bacteria*		
	None**(n = 9)	Other**bacteria (n = 5)	*Gardnerella *biofilm (n = 5)	None**(n = 17)	Other**bacteria (n = 8)	*Gardnerella *biofilm (n = 2)
*Gardnerella *biofilm (n = 14)	1 (12%)	2 (25%) 30	5 (62%) 3810	2 (33%)	2 (33%) 22	2 (33%) 35
No *Gardnerella *biofilm (n = 32)	8 (73%)	3 (27%)	0	15 (71%)	6 (29%)	0

Numbers in *italics* are the mean concentrations of bacteria ×10^9^/ml found in tissues of women positive for bacteria.

Percentages represent the numbers of women with bacteria detected on the endometrium in subgroups, i.e. with and without bacterial vaginosis and with or without missed abortion.

**Table 2:** Occurrence of bacteria on fallopian tube epithelium in women with and without bacterial vaginosis.

*Presence of bacterial vaginosis*	*Presence of bacteria on the fallopian tube epithelium*					
	*Extra-uterine pregnancy (n = 1)*			*Other indications (n = 21)*		
	*fallopian tube bacteria*			*fallopian tube bacteria*		
	None**(n = 0)	Other**bacteria (n = 0)	*Gardnerella *biofilm (n = 1)	None (n = 17)	Other**bacteria (n = 4)	*Gardnerella *biofilm (n = 0)
*Gardnerella* vaginal**biofilm (n = 4)	0	0	1 (100%)	3 (100%)	0	0
No *Gardnerella* vaginal**biofilm (n = 18)	0	0	0	14 (78%)	4 (22%)4	0

Numbers in *italics* show mean concentrations of bacteria x 10^9^/ml found in tissues of women positive for bacteria.

Percentages represent the numbers of women with bacteria detected on the endometrium in subgroups, i.e. with and without bacterial vaginosis and with or without missed abortion.

**Figure 2:**
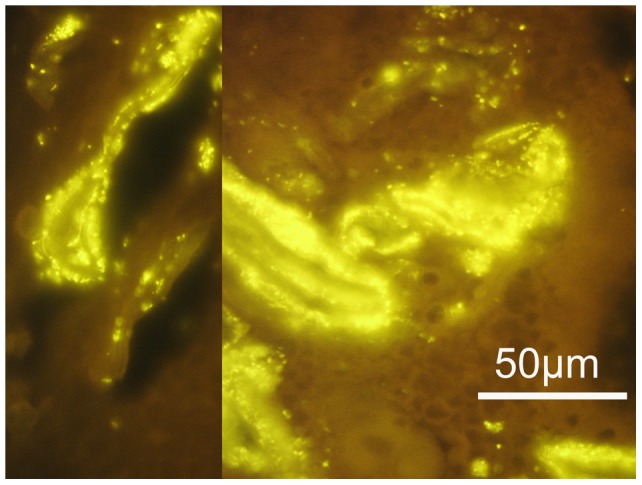
*Gardnerella* dominated polymicrobial biofilm attached to the endometrium. Prolific *Gardnerella* dominated biofilm within follicular (left panel) and luteal (right panel) endometrium. Hybridization is performed with *Gard C3* orange fluorescence FISH probe, at magnification ×1000. The anatomic location of bacteria within the endometrium excludes the possibility of contamination.

**Figure 3:**
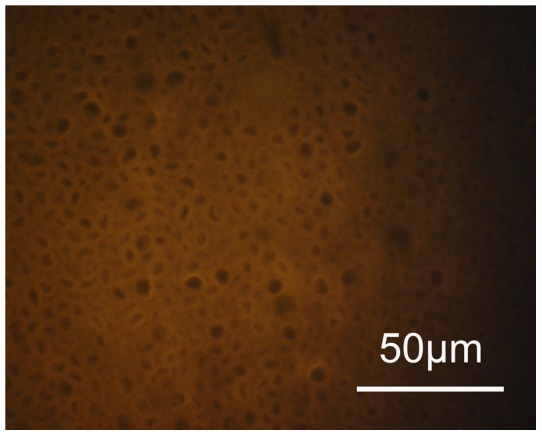
Endometrial sample free of bacteria. An example of negative hybridization with the universal Eub 338-C3 probe with no bacteria on the endometrium showing fluoresence (magnification ×1000).

**Figure 4:**
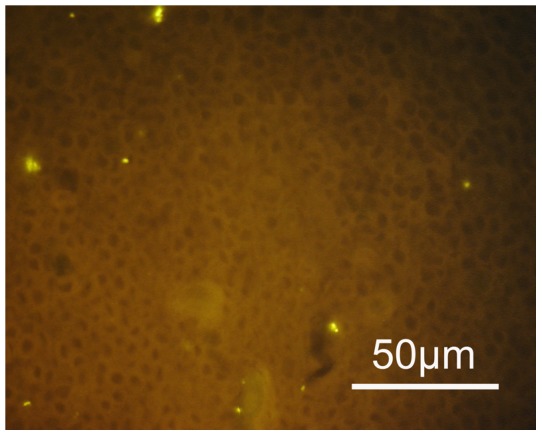
Bacteria other than *Gardnerella* colonising the endometrial epithelium. Singular bacteria positively hybridize with the Eub 338 -C3 probe specific for all bacteria are located on the endometrium (orange fluorescence magnification ×1000). These bacteria are negative, when hybridized with the *Gardnerella* probe, never build biofilms and are “planktonic”.

A structured polymicrobial *Gardnerella vaginalis* biofilm attached to the endometrial tissue was found in five of eight pregnant women undergoing curettage and in two of the six non-pregnant women having curettage (see Figure 2). A structured polymicrobial *Gardnerella vaginalis* biofilm on the fallopian tube epithelium was found in one of the four women with bacterial vaginosis undergoing fallopian tube resection for extra-uterine pregnancy. In none of the women without bacterial vaginosis was a structured polymicrobial *Gardnerella vaginalis* biofilm found in the endometrial or fallopian tissue (see Figure 3). Bacteria, other than *Gardnerella*, covering the uterine or fallopian epithelium (see Figure 4), were found in four women having bacterial vaginosis (4/18) and in 13 women not having bacterial vaginosis (13/50) (see Table 1 and Table 2).

Accordingly, women who had evidence of a vaginal bacterial vaginosis biofilm had a 50.0% (95% CI 24.0–76.0) risk of presenting with an endometrial *Gardnerella vaginalis* biofilm. Overall, compared to women not having bacterial vaginosis (13/50), women with bacterial vaginosis (12/18) had a significantly increased risk of bacterial colonization of the endometrium and fallopian tubes with an odds ratio of 5.7 (95% CI, 1.8–18.3, *P* = 0.002) (see Table 1, Table 2, and Table 3).

**Table 3:** Occurrence of bacteria on the endometrium or fallopian tube epithelium in women with and without a vaginal structured polymicrobial *Gardnerella* biofilm – overview.

	Presence of bacteria in uterus or tubes
Absence of a vaginal *Gardnerella* biofilm (n = 50)	13/50 (26.0%)
Presence of a vaginal *Gardnerella* biofilm (n = 18)-non-pregnant**women (n = 9)-pregnant women (n = 9)	12/18 (67.0%) 4/9 (44.0%) 8/9 (89.0%)

In a preliminary predictive model it was found that both pregnancy/missed abortion (AOR  = 41.5, 95% CI 5.0–341.9, *P*<0.001) and the presence of bacterial vaginosis (AOR  = 23.2, 95% CI 2.6–205.9, *P*<0.001) were highly predictive of the presence of uterine or fallopian bacterial colonisation when compared to non-pregnant women not having bacterial vaginosis.

## Discussion

We documented that in patients having curettage for missed abortion, half of the women having bacterial vaginosis were found to have a bacterial vaginosis biofilm covering the endometrium. In addition, we found that patients having bacterial vaginosis were significantly more likely to show upper genital tract colonisation with BV-associated and other bacteria compared to women not having bacterial vaginosis.

Traditionally, the endometrial cavity has been considered a sterile upper genital tract compartment. Several studies have challenged this view and found high rates of uterine bacterial colonization [10], [12]. These studies may be thought to be due to contamination from the vagina. However, microorganisms have been recovered from the endometrial cavity of non-pregnant women after hysterectomy under conditions that minimize the risk of specimen contamination [13]–[16] and from samples obtained by transfundal aspiration of the endometrial cavity in postpartum patients undergoing tubal ligation [17]. Hence, the view of the endometrial cavity as a permanently sterile body compartment may not be longer tenable [18]. As we and others have previously shown through the use of molecular, culture-independent techniques, that the vagina, especially in the setting of bacterial vaginosis, harbours a vast number of bacterial species that were previously unrecognized [19]–[21], with the full extent of uterine microbiome to be determined.

In the present study, we used a culture-independent, molecular targeting 16 s rDNA-based approach for bacterial identification whereby FISH-based visualization offers some kind of microscale imaging technique. A structured polymicrobial *Gardnerella vaginalis* biofilm was found in the uterus in about half of the patients having a missed abortion and bacterial vaginosis and even in a fallopian tube in one out of four patients undergoing a salpingectomy while having bacterial vaginosis. It appears that bacterial vaginosis of the vagina can be accompanied by a bacterial vaginosis biofilm in the upper genital tract.

While the clinical significance of planktonic bacteria in the uterine cavity has been questioned, the presence of full-blown uterine bacterial vaginosis – possibly better termed *gardnerellosis* – may be of major importance to the morbidity associated with bacterial vaginosis and adverse pregnancy outcome in particular. From our observations in the vagina, we know that the biofilm can be present for prolonged periods of time without causing symptoms [7], [11]. The same might be true for the endometrial biofilm; although one might also speculate that in the absence of pregnancy the biofilm might be released and expelled by menstruation.

How then, could the biofilm be of importance to infection-mediated foetal loss or preterm birth? A possible explanation suggested by Espinoza *et al*, is that microorganisms normally present on the endometrial surface do not elicit a proinflammatory response during pregnancy and that this allows for pacific coexistence of the pregnancy along with microorganisms, however that such innocuous cohabitation may end if the host (mother/conceptus or both) recognizes the microorganisms using pattern recognition receptors, and launches a proinflammatory response [18]. Espinoza *et al* further suggested that during pregnancy changes in the virulence patterns (e.g. release of planktonic bacteria from biofilms) could also alter the balance and lead to infection/inflammation-induced preterm birth [18]. If anything, the presence of a tremendous reservoir of potentially pathogenic bacteria stacked in a biofilm configuration adhering to the endometrium in the immediate vicinity of the myometrium and of the amniotic compartment may be a key element to the pathogenesis of adverse pregnancy outcome.

It must be acknowledged that the significance of our observations primarily relates to the demonstration of an endometrial polymicrobial biofilm. The preliminary inferences on the association with pregnancy however may be biased, since patients in our cohort were primarily patients with missed abortion. It may further be added, that it is highly unlikely that our observations may have been biased by vaginal contamination, considering the biofilm strongly adheres to the endometrial and vaginal tissues. It is further of note, that about one in four patients also presented with high concentrations (<10^9^) of endometrial bacteria that are not part of the typical bacterial vaginosis microbiota (see Table 1 and Table 2).

It may be concluded that further study is warranted to disentangle the intricate relationship between the vaginal microbiota and adverse pregnancy outcome; however such investigations might now also focus on the presence of a *Gardnerella vaginalis* biofilm in the upper genital tract.
